# CdgB Regulates Morphological Differentiation and Toyocamycin Production in *Streptomyces diastatochromogenes* 1628

**DOI:** 10.3390/ijms25073878

**Published:** 2024-03-30

**Authors:** Rui Wang, Zixuan Zhang, Xiaoping Yu, Yang Song, Xuping Shentu

**Affiliations:** 1 Zhejiang Provincial Key Laboratory of Biometrology and Inspection and Quarantine, College of Life Science, China Jiliang University, Hangzhou 310018, Chinayxp@cjlu.edu.cn (X.Y.); 2 Department of Chemistry, Zhejiang University, Hangzhou 310027, China

**Keywords:** CdgB, toyocamycin, *Streptomyces diastatochromogenes* 1628, morphological differentiation, secondary metabolism

## Abstract

Bis (3′,5′)-cyclic diguanylic acid (c-di-GMP) is a ubiquitous second messenger that controls several metabolic pathways in bacteria. In *Streptomyces*, c-di-GMP is associated with morphological differentiation, which is related to secondary metabolite production. In this study, we identified and characterized a diguanylate cyclase (DGC), CdgB, from *Streptomyces diastatochromogenes* 1628, which may be involved in c-di-GMP synthesis, through genetic and biochemical analyses. To further investigate the role of CdgB, the *cdgB*-deleted mutant strain Δ-*cdgB* and the *cdgB*-overexpressing mutant strain O-*cdgB* were constructed by genetic engineering. A phenotypic analysis revealed that the O-*cdgB* colonies exhibited reduced mycelium formation, whereas the Δ-cdgB colonies displayed wrinkled surfaces and shriveled mycelia. Notably, O-*cdgB* demonstrated a significant increase in the toyocamycin (TM) yield by 47.3%, from 253 to 374 mg/L, within 10 days. This increase was accompanied by a 6.7% elevation in the intracellular concentration of c-di-GMP and a higher transcriptional level of the *toy* cluster within four days. Conversely, Δ-*cdgB* showed a lower c-di-GMP concentration (reduced by 6.2%) in vivo and a reduced toyocamycin production (decreased by 28.9%, from 253 to 180 mg/L) after 10 days. In addition, *S. diastatochromogenes* 1628 exhibited a slightly higher inhibitory effect against *Fusarium oxysporum* f. sp. *cucumerinum* and *Rhizoctonia solani* compared to Δ-*cdgB*, but a lower inhibition rate than that of O-*cdgB*. The results imply that CdgB provides a foundational function for metabolism and the activation of secondary metabolism in *S. diastatochromogenes* 1628.

## 1. Introduction

**Figure 1:**
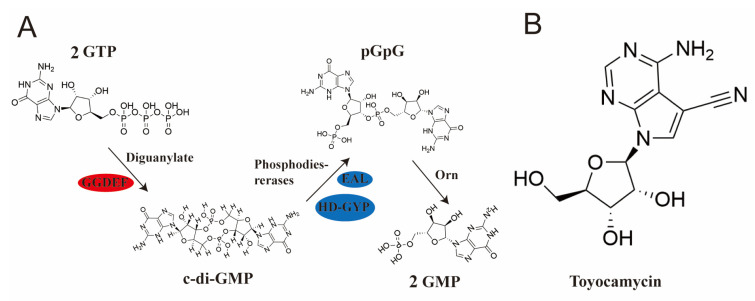
The pathways for the biosynthesis and catabolism of c-di-GMP and the chemical structure of toyocamycin. (**A**) The pathways for the biosynthesis and catabolism of c-di-GMP. Gly-Gly-Asp-Glu-Phe (GGDEF), Glu-Ala-Leu (EAL), [His-Asp]-[Gly-Tyr-Pro] (HD-GYP), Oligoribonuclease (Orn). (**B**) The chemical structure of toyocamycin.

*Streptomyces diastatochromogenes* 1628 is an important Gram-positive bacterium that is used to produce several kinds of antibiotics, including toyocamycin, tetramycin A, tetramycin P, and tetrin B, which are widely used in the fields of medicine, agriculture, and food [1,2,3,4]. Among them, toyocamycin, an alkaline nucleoside antibiotic (Figure 1B), exhibits antibacterial and antifungal activities [5]. However, the production of toyocamycin by wild-type *S. diastatochromogenes* 1628 (Wild 1628) was only 152.1 mg/L after 84 h; thus, its use in industrial applications remains limited [6]. To enhance toyocamycin production, the related transcriptional regulators ToyA [6] and AdpA [7] were overexpressed to activate the toyocamycin synthesis gene cluster, and the toyocamycin yield increased by 80% and 120.1% in *S. diastatochromogenes* 1628, respectively. However, the production still cannot meet the demand for toyocamycin due to the intricate regulatory networks and complex morphological transitions inherent to *Streptomyces* metabolism.

The growth of *Streptomyces* involves complex life cycles that include distinct vegetative growth, aerial mycelium formation, and sporulation phases [8]. These developmental transitions are tightly regulated to ensure the production of various bioactive compounds, including antibiotics. The *bld* and *whi* genes have been identified as pivotal developmental regulators; the Bld regulatory factor controls the formation of the aerial mycelium and the Whi regulator has a significant impact on spore development in *Streptomyces* [9]. Therefore, we suggested that Bld and Whi regulate the production of toyocamycin in *S. diastatochromogenes* 1628.

Furthermore, bis (3′,5′)-cyclic diguanylic acid (c-di-GMP) is a significant signal molecule that regulates a variety of fundamental physiological and morphological differentiation processes. It is widely found in bacteria, actinomycetes, fungi, and so on. C-di-GMP enhances BldD binding to target DNA [10]. Strikingly, BldD encodes a DNA-binding protein with the typical HTH structural domain, and the target genes of BldD encode regulatory proteins in *S. coelicolor* [11]. Additionally, c-di-GMP controls reproductive hyphal spore differentiation by activating the binding of the anti-σ factor (RsiG) to the *Streptomyces*-specific σ factor (σ^WhiG^) [12]. Therefore, c-di-GMP is the key regulator in morphological development and antibiotic production.

C-di-GMP is formed by the cyclization of two molecules of guanosine triphosphate (GTP) catalyzed by diguanylate cyclase (DGC). Conversely, phosphodiesterases (PDEs) hydrolyze c-di-GMP into either one 5′-phosphoguanylyl-(3′,5′)-guanosine (pGpG) or two 5′-guanylic acid (GMP) molecules (Figure 1A) [13,14,15]. The intracellular levels of c-di-GMP in mycobacteria are regulated by DGCs containing a Gly-Gly-Asp-Glu-Phe (GGDEF) motif, while the PDEs contain a Glu-Ala-Leu (EAL) or [His-Asp]-[Gly-Tyr-Pro] (HD-GYP) motif. In a previous study, moenomycin A (MmA) production was found to be increased when *cdgB_gh_* encoding a DGC was overexpressed, while the effect was reversed when *cdgB_gh_* was deleted in *Streptomyces ghanaensis*. Furthermore, the inactivation of *rmdB_gh_*, which encodes a PDE, resulted in a significant increase in MmA production [16]. However, the impact of CdgB on other secondary metabolites, particularly nucleoside antibiotics, remains to be elucidated.

In this study, the morphological effect of CdgB on growth and mycelial morphology was investigated by the overexpression and deletion of *cdgB* in *S. diastatochromogenes* 1628. In addition, the positive effect of CdgB on toyocamycin production was evaluated by the yield and a transcriptional analysis. Furthermore, the influence of CdgB on the antimicrobial properties of fermentation broths of *Fusarium oxysporum* f. sp. *cucumerinum* and *Rhizoctonia solani* was assessed by inhibition zones. These results have the potential to inform strategies for improving toyocamycin production, thus contributing to the broader field of antibiotic discovery and production.

## 2. Results

### 2.1. The Analysis of Phylogenetic Trees for CdgB and CdgB’s Function in c-di-GMP Synthesis in S. diastatochromogenes 1628

**Figure 2:**
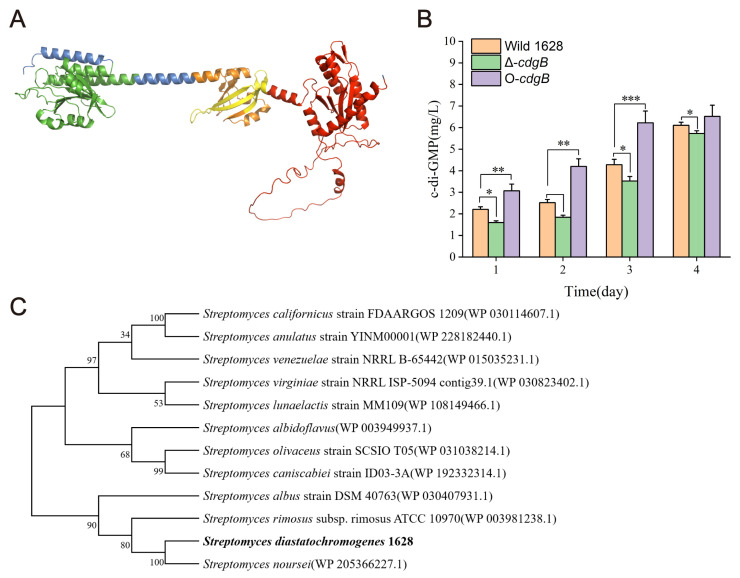
A bioinformatics analysis of CdgB in *S. diastatochromogenes* 1628 and the effects of CdgB on concentrations of c-di-GMP. (**A**) The three-dimensional (3D) structural models of CdgB in PyMOL. The GAF domain is highlighted in green, the PAS domain is highlighted in orange, the PAC domain is highlighted in yellow, and the GGDEF domain is highlighted in red, the rest is blue, using the AlphaFold model A0A125NTF3 as the template, with 87.79% identification. (**B**) Intracellular c-di-GMP concentrations of the Wild 1628, Δ-*cdgB*, and O-*cdgB* strains cultivated in fermentation medium. (**C**) Phylogenetic analyses of CdgB and its homologs. CdgB of *S. diastatochromogenes* 1628 is highlighted in bold. * indicates the significance value (* *p* < 0.05, ** *p* < 0.01, *** *p* < 0.001).

The *cdgB* genes were identified from SRIM_RS24680 sequences, and the *cdgB* in *S. diatatochromogenes* 1628 was 100% similar to the SRIM_RS24680 sequences. CdgB consists of 563 amino acids and has a molecular weight of 60.6 kDa in *S. diastatochromogenes* 1628. CdgB has a GAF-PAS-PAC signaling domain and a GGDEF domain (Figure 2A). The GAF domain binds to cyclic mononucleotides [17], the PAS domain is involved in the redox potential [18], the PAC domain is involved in glycolysis, and the GGDEF domain is involved in c-di-GMP synthesis [19]. To explore the role of CdgB in *S. diastatochromogenes* 1628, phylogenetic trees were constructed by analyzing homologous protein coding sequences from diverse *Streptomyces* genomes through bioinformatics methods (Figure 2C). The amino acid sequence of CdgB in *S. diastatochromogenes* 1628 was found to be exactly the same as the homologous protein in *Streptomyces noursei*.

As CdgB has been shown to regulate c-di-GMP synthesis in *S. coelicolor* [19] and *S. venezuelae* [20], we investigated its influence on c-di-GMP synthesis in *S. diastatochromogenes* 1628. Subsequently, we examined the c-di-GMP levels in Wild 1628, Δ-*cdgB* (*cdgB* deletion mutant), and O-*cdgB* (*cdgB* overexpression strain) over a period of four days. Compared with Wild 1628, O-*cdgB* exhibited a significantly elevated concentration of c-di-GMP during the first three days (Figure 2B), reaching 6.52 mg/L on the fourth day. However, it is noteworthy that the concentration of c-di-GMP did not show significant differences on the fourth day, suggesting the presence of phosphodiesterases (PDEs) that may degrade c-di-GMP to prevent excessive accumulation in vivo. Conversely, the concentration of c-di-GMP in Δ-*cdgB* was significantly reduced throughout the four-day period, with a notable decrease of 6.2% compared to that of Wild 1628, reaching 5.73 mg/L on the fourth day. These results strongly support the role of CdgB as a critical diguanylate cyclase (DGC) responsible for c-di-GMP synthesis.

### 2.2. Effect of CdgB on the Morphological Characteristic of S. diastatochromogenes 1628

**Figure 3:**
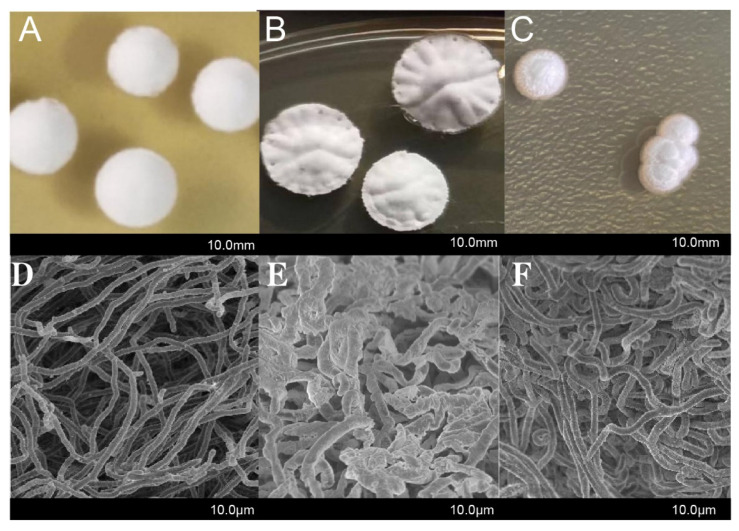
Morphological analysis of Wild 1628, Δ-*cdgB*, and O-*cdgB* strains. (**A**) Wild 1628 colony morphology. (**B**) Δ-*cdgB* colony morphology. (**C**) O-*cdgB* colony morphology. Scanning electron micrographs of Wild 1628 (**D**), Δ-*cdgB* (**E**), and O-*cdgB* (**F**).

To examine whether the deletion or overexpression of *cdgB* affects the morphological development of *S. diastatochromogenes* 1628, we conducted an experiment using Wild 1628, Δ-*cdgB*, and O-*cdgB*. These strains were cultivated on a GYM plate medium at 28 °C. The Wild 1628 colonies exhibited a round shape with smooth surfaces, and the mycelium appeared flat and smooth (Figure 3A,D). O-*cdgB* displayed a reduced amount of mycelium (Figure 3C,F), a phenomenon that has been previously observed in *S. coelicolor*, where the overexpression of *cdgB* and *cdgD* inhibited the formation of an aerial mycelium [19,21]. Δ-*cdgB* showed a slightly raised center, thicker colonies, and a shriveled mycelium and produced more mature spores (Figure 3B,E), which was similar to the phenotype obtained by the deletion of *cdgB* in *S. venezuelae* [22]. These findings suggest that CdgB plays a crucial role in regulating the morphological development of *S. diastatochromogenes* 1628.

### 2.3. Effect of CdgB on Toyocamycin Production

**Figure 4:**
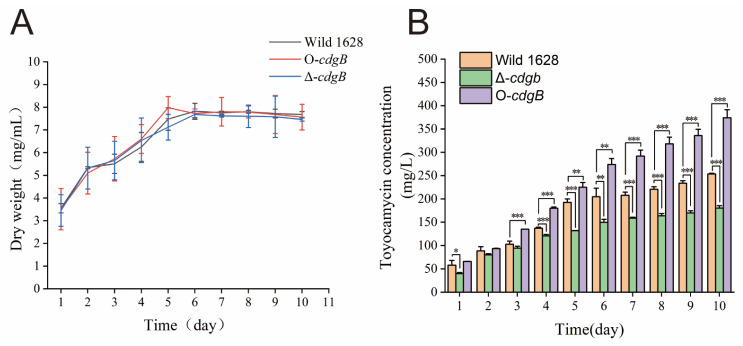
Effects of CdgB on cell growth and toyocamycin production. (**A**) Growth curves of Wild 1628, Δ-*cdgB*, and O-*cdgB* strains cultured in fermentation medium. Biomass was evaluated as dry cell weight. (**B**) Quantitative toyocamycin production of Wild 1628, Δ-*cdgB*, and O-*cdgB* cultured in fermentation medium. * indicates the significance value (* *p* < 0.05, ** *p* < 0.01, *** *p* < 0.001).

*S.diastatochromogenes* 1628 was used as a control to compare the level of cell growth of Δ-*cdgB* and O-*cdgB*. As shown in Figure 4A, no significant effect on cell growth was observed for Δ-*cdgB* and O-*cdgB* compared to Wild 1628.

To investigate the effect of CdgB on toyocamycin production, the Wild 1628, Δ-*cdgB*, and O-*cdgB* strains were cultured in GYM medium and fermentation broths were collected at different time intervals for an HPLC analysis. As shown in Figure 4B, the toyocamycin production remained similar among the strains during the initial 1–3 days of fermentation, and then the toyocamycin yield started to show significant differences from day 4 to day 10. The TM production in O-*cdgB* was higher than in Wild 1628, and the highest titer reached 374 mg/L by the tenth day, which was 47.3% higher than that in Wild 1628. Conversely, the TM production in Δ-*cdgB* was lower than in Wild 1628, and the highest titer (180 mg/L) was 28.9% lower than that of Wild 1628. The results showed that an alteration in the intracellular c-di-GMP levels had a major impact on antibiotic production, with the overexpression of *cdgB* enhancing toyocamycin production and its deletion exerting the opposite effect.

### 2.4. The Effect of CdgB on Toy Cluster Expression Level

The life cycle of *Streptomyces* is critical for morphological transitions, which typically occur during the first four days. In addition, significant variations in the concentration of c-di-GMP are observed within this timeframe, leading to probable changes in the transcriptional levels. To examine the effect of CdgB on the transcription of the *toy* clusters, quantitative real-time RT-PCR (qRT-PCR) was applied to analyze the transcription levels of the *toy* genes (*toyA*, *toyB*, *toyC*, *toyD*, *toyE*, *toyF*, *toyG*, *toyH*, *toyI*, and *toyM*). The total RNAs were prepared from Wild 1628, Δ-*cdgB*, and O-*cdgB* in a fermentation medium and used as templates for cDNA synthesis and the qRT-PCR analysis.

**Figure 5:**
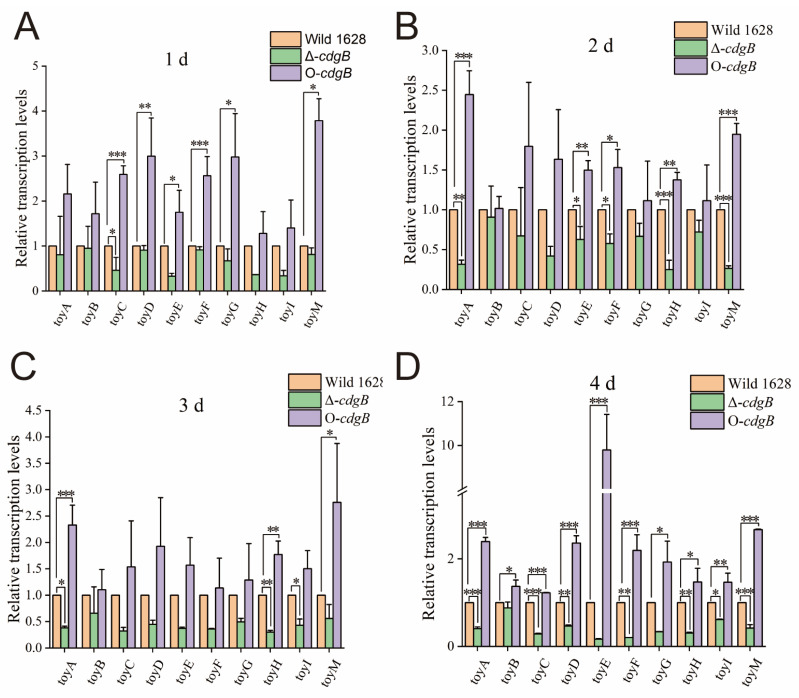
Transcription of *toy* gene clusters over 4 days. Comparison of transcriptional levels of *toyA* as well as toy genes (*toyB*~*toyM*) involved in TM biosynthesis pathway by using quantitative reverse-transcription PCR in Wild 1628, Δ-*cdgB*, and O-*cdgB* on the first day (**A**), second day (**B**), third day (**C**) and forth day (**D**). * indicates the significance value (* *p* < 0.05, ** *p* < 0.01, *** *p* < 0.001).

A comparison with Wild 1628 revealed a substantial decrease in the transcriptional levels of the toy clusters in Δ-*cdgB*. In contrast, the transcriptional levels of the *toy* cluster of O-*cdgB* were obviously increased compared with those of Wild 1628 (Figure 5). These findings suggest that the overexpression of *cdgB* enhances the transcript levels of all the biosynthetic genes of TM and further improves the TM production in O-*cdgB*. The increased production of secondary metabolites may be attributed to the elevated intracellular c-di-GMP levels resulting from CdgB gene overexpression.

### 2.5. Effect of CdgB on the Inhibitory Activity of S. diastatochromogenes 1628

**Figure 6:**
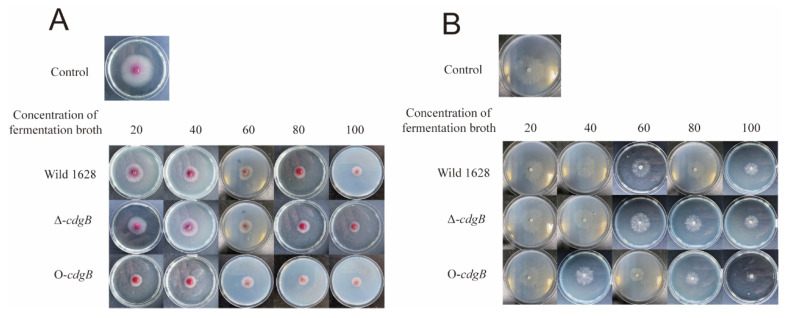
Fermentation broth inhibited growth of *Fusarium oxysporum* f. sp. *cucumerinum* and *Rhizoctonia solani*. (**A**) Inhibitory effect of fermentation broths on *Fusarium oxysporum* f. sp. *cucumerinum*. (**B**) Inhibitory effect of fermentation broths on *Rhizoctonia solani*.

*Streptomyces* demonstrates remarkable antimicrobial activity with a broad spectrum against diverse phytopathogenic fungi. The fermentation broth effectively suppressed the growth of *Fusarium oxysporum* f. sp. *cucumerinum* and *Rhizoctonia solani* in a concentration-dependent manner. As shown in Figure 6, a gradual increase in the concentration of the fermentation broth led to increased inhibitory effects on *Fusarium oxysporum* f. sp. *cucumerinum* and *Rhizoctonia solani*. When comparing the inhibitory ability, Wild 1628 exhibited a slightly higher inhibitory effect against the phytopathogenic fungi in comparison to Δ-*cdgB*, but a lower inhibition effect than that of O-*cdgB*. Therefore, the overexpression of *cdgB* enhances the inhibition of phytopathogenic fungi by *S. diastatochromogenes* 1628.

## 3. Discussion

C-di-GMP is a ubiquitously reported secondary messenger that plays a regulatory role in cell motility, biofilm formation, and dispersion [23]. Moreover, c-di-GMP is involved in morphological differentiation [20] and secondary metabolite production [16] in *Streptomyces* sp. Diguanylate cyclases (DGCs) and phosphodiesterases (PDEs) have been extensively studied in *S. coelicolor* [21,24] and *S. venezuelae* [20]. We identified the *cdgB* gene encoding DGCs in *S. diastatochromogenes* 1628. A bioinformatics analysis revealed that CdgB possesses both a GAF-PAS-PAC signaling domain and a GGDEF domain, which is consistent with the motif organization of CdgB in *S. venezuelae* and *S. coelicolor*.

During morphological differentiation, we observed that O-*cdgB* inhibited the formation of an aerial mycelium, a phenomenon that was also observed in *S. coelicolor* upon the overexpression of *cdgB* and *cdgD* [19,21]. Furthermore, colonies of the Δ-*cdgB* mutant exhibited elaborate radial ridges on their surface, resembling the phenotypes observed in *S. venezuelae* upon the deletion of *cdgB* [22]. The reason for this phenomenon may be due to the concentration of c-di-GMP. The dissociation of the BldD-(c-di-GMP) complex at low c-di-GMP levels leads to the depression of BldD targets and allows for the formation of an aerial mycelium. The BldD-(c-di-GMP) complex at high c-di-GMP levels results in the repression of BldD targets and the formation of an aerial mycelium.

Next, it was found that intracellular c-di-GMP levels exert a significant influence on antibiotic production. In previous studies, a dimer formed by c-di-GMP and BldD was effective in inhibiting *wblA* transcription, positively regulating *adpA*, and stimulating the synthesis of moenomycin A (MmA) in *Streptomyces ghanaensis* [16]. AdpA serves as a crucial regulator of secondary metabolism and morphological differentiation [25], governing the expression of over 500 genes in *Streptomyces griseus* [26]. Our previous studies have demonstrated that AdpA positively modulates the expression of the toy gene cluster, thereby enhancing toyocamycin production in *S. diastatochromogenes* 1628 [7]. In this study, we found that the concentration of c-di-GMP was positively correlated with the yield of toyocamycin. The overexpression of *cdgB* resulted in elevated toyocamycin production and enhanced bacterial suppression in *Fusarium oxysporum* f. sp. *Cucumerinum* and *Rhizoctonia solani*, whereas the deletion of the *cdgB* strains showed the opposite effect. This phenomenon may be attributed to the enhanced formation of c-di-GMP-BldD dimers, which positively regulate AdpA and stimulate toyocamycin synthesis. This mechanism is analogous to that described by Makitrynskyy et al. [16], who reported that BldD directly activates *adpA* transcription, thereby influencing secondary metabolite production and morphological regulation. In addition, the qRT-PCR analysis revealed that the transcriptional level of the toy genes was upregulated in O-*cdgB* and downregulated in Δ-*cdgB*. These results suggest that c-di-GMP affects the expression of the *toy* cluster though the c-di-GMP-BldD and AdpA pathway. In conclusion, these results provide a useful approach for improving toyocamycin production.

## 4. Materials and Methods

### 4.1. Strains, Plasmids, Primers, and Culture Conditions

The *Streptomyces*, *E. coli*, fungi, and plasmids used for this study are listed in Table 1. The primers are listed in Supplementary Table S1. *S. diastatochromogenes* 1628 has been deposited in the China General Microbiological Culture Collection (CGMCC 2060), and was isolated from Tianmu Mountain, Zhejiang Province, in previous work [27]. The strain was grown at 28 °C in a glucose yeast mineral (GYM) medium [28] for a morphological analysis. Toyocamycin production was carried out using the same media and methods as previously reported [27]. *Fusarium oxysporum* f. sp. *cucumerinum* (CICC 2532) and *Rhizoctonia solani* (CICC 40529) have been deposited in the China Center of Industrial Culture Collection as experimental strains for fungal suppression. *E. coli* JM109 and pIB139 were purchased from Sangon Biotech (Shanghai) Co., Ltd., Shanghai, China, *E. coli* ET12567(PUZ8002) was purchased from Wuhan Miaoling Biotechnology Co., Ltd., Wuhan, China.

### 4.2. The Phylogenetic Analysis of CdgB

Multiple sequence alignment and molecular phylogeny analyses were performed using the MEGA 11 software [29]. NCBI’s protein basic local alignment search tool (Blastp) program was used to assess the CdgB similarity levels by comparing protein sequences. The evolutionary history was inferred using the neighbor-joining method. A bootstrap analysis on 1000 replicates was utilized to investigate the stability of the evolutionary branches in the tree.

### 4.3. Construction of cdgB Disruption and Overexpression Mutants

The disruption of *cdgB* was performed by gene replacement via homologous recombination, as described by Jiang et al. [30]. To construct the *cdgB* disruption mutant (Δ-*cdgB*), the upstream and downstream homology arms (~2 kb each) of the *cdgB* gene were amplified with the primer pairs *cdgB*LF/*cdgB*LR and *cdgB*RF/*cdgB*RR, using *S. diastatochromogenes* 1628 genomic DNA as a template (Table S1). The respective 3′ and 5′ ends of the upstream and downstream fragments shared 40 bp complementary regions. The upstream and downstream homology arms were ligated by overlap extension PCR with the primers *cdgB*LF and *cdgB*RR (Table S1). Then, the linkage fragments were inserted into the *Hind* III and *Eco*RV sites of pKC1139 to yield pKC1139-*cdgB* and pKC1139-*cdgB*; these were then introduced into *E. coli* JM109 for amplification. The recombinant plasmids were isolated and used for the transformation into *E. coli* ET12567/PUZ8002, and they were then transferred into *S. diastatochromogenes* 1628 via conjugation to obtain recombinant strains containing apramycin resistance. Then, the selected cells were cultured on apramycin-resistant (Apr^R^) plates at 37 °C for homologous recombination to obtain single-exchange strains, followed by a relaxation culture at 28 °C in non-resistant plates to obtain double-exchange strains. These double-exchange strains were screened on apramycin-resistant and non-resistant plates to select the non-resistant strains. The strains that did not grow on the resistant plates and grew on the non-resistant plates were picked for PCR verification. The *cdgB* deletion strain was confirmed by PCR using the primers *cdgB*LF and *cdgB*RR, and was designated as Δ-*cdgB*.

For the construction of the *cdgB* overexpression mutant (O-*cdgB*), the *cdgB* open reading frames (ORFs) were amplified by PCR with the primers O-*cdgB*-F and O-*cdgB*-R (Table S1), using *S. diastatochromogenes* 1628 genomic DNA as a template. The PCR product was inserted into the *Nde*I and *Eco*RV sites of pIB139 to yield pIB139-*cdgB*, and pIB139-*cdgB* was introduced into *E. coli* JM109 for amplification. The recombinant plasmids were isolated and used for the transformation into *E. coli* ET12567/PUZ8002, and they were then transferred into *S. diastatochromogenes* 1628 via conjugation to obtain recombinant strains containing apramycin resistance [31]. The genotype of mutants was confirmed by PCR and sequencing. The recombinant *S. diastatochromogenes* 1628 containing overexpressed *cdgB* was named O-*cdgB.*

### 4.4. Morphological Assessment

To analyze the morphological changes in the strains, *S. diastatochromogenes* 1628, Δ-*cdgB*, and O-*cdgB* were cultured in GYM agar at 28 °C and photographed after waiting for multiple single colonies to grow.

For the SEM analysis, 1 mL of fermentation solution of the Wild 1628, Δ-*cdgB*, or O-*cdgB* strains that had been incubated for two days at 28 °C in GYM medium was taken. The mycelia were collected through centrifugation and washed twice with phosphate-buffered saline (PBS), and a fixative solution of 2.5% glutaraldehyde was slowly applied to the tube wall. After that, the tube was stored in a refrigerator at 4 °C. The bacterial morphological characteristics of *S. diastatochromogenes* 1628, Δ-*cdgB*, and O-*cdgB* were observed using scanning electron microscopy (SEM) (SU3800, Hitachi, Tokyo, Japan).

### 4.5. The Effect of cdgB on Cell Growth and Toyocamycin Production

The wild-type, Δ-*cdgB*, and O-*cdgB* strains were cultured in GYM medium for two days as seed, 1 mL of the cultured seed was transferred into GYM medium, and 2 mL of the cell culture was taken at intervals of 24 h. The fermentation broth was obtained by centrifuging at 10,000 rpm for 5 min, filtered through a 0.22 μm microporous membrane, and then analyzed by HPLC. The yield of toyocamycin was assayed with reference to Ma et al. [32]. The dry weight was obtained by collecting 60 mL of cell culture, centrifuging to remove the supernatant, and drying at 108 ℃ to a constant weight.

### 4.6. Intracellular c-di-GMP Extraction and Detection

*Streptomyces* samples were collected after being cultured for 1 day, 2 days, 3 days, or 4 days in GYM medium. The *Streptomyces* samples were centrifuged at 10,000 rpm and the strains were collected and frozen in liquid nitrogen, and then ground into fine powder. Then, the powder was incubated with 2 M formic acid in ice-cold water for 30 min, followed by the addition of ammonium acetate (50 mM, pH of 4.5). The supernatants were harvested by centrifugation at 5000× *g* for 10 min at 4 °C. The solid-phase extraction column (Welch) was prepared by adding 1 mL of methanol (MeOH) and 1 mL of 50 mM ammonium acetate (NH_4_OAC) (pH of 4.5), followed by the addition of supernatant and washing of the column with 1 mL of 50 mM NH_4_OAC (pH of 4.5) and 1 mL of MeOH. The c-di-GMP in the SPE column was eluted with 1 mL of a methanol/H_2_O/ammonium hydroxide (20:70:10) solution. The collected liquid was blown dry with nitrogen, subsequently dissolved in 200 µL of water, and filtered through a 0.2 µm filter (Agilent ValueLab) into a collection tube. The samples were analyzed using an ACQUITY UPLC I-Class ultra-high-performance liquid chromatography (HPLC) system and a Xevo TQ-S mass spectrometer (Milford, MA, USA) using a CORTECS UPLC T3 column (Waters, 1.6 μm 2.1 × 150 mm). The c-di-GMP analysis was determined according to the method described by Makitrynskyy et al. [16].

### 4.7. The Transcriptional Level of Toy Clusters

The mycelia of Wild 1628, Δ-*cdgB*, and O-*cdgB* were collected after being cultured for 1 day, 2 days, 3 days, or 4 days in GYM medium. The mycelium was transferred onto sterile filter paper to absorb water, and then ground into a powder using liquid nitrogen. The extraction of RNA was conducted by One Step PrimeScript^™^ III RT-qPCR Mix (Takara, Dalian, China), and cDNA first-strand synthesis was performed using a PrimeScript^™^ RT reagent kit with gDNA Eraser (Takara, Dalian, China). The primers are listed in Table S1 and qRT-PCR was performed as described previously [7]. The *gyrB* of *S. diastatochromogenes* 1628 was used as an internal control gene to normalize the strain amount.

### 4.8. The Antimicrobial Activity against Filamentous Fungi

The wild-type, Δ-*cdgB*, and O-*cdgB* strains were grown in GYM medium as seeds for two days and 1 mL of the cultured seeds was added to GYM medium for four days to obtain the fermentation broth. The fermentation broth was filtered through a 0.22 µm sterile filter and diluted to different concentrations of 20%, 40%, 60%, or 80%. A total of 100 µL of fermentation broth was added to potato dextrose agar (PDA). An equal amount of water was used as a control, and 5 mm diameter filamentous fungi were obtained with a hole punch and placed on the plate’s center. The crosshatch method was used to measure the growth diameter of the fungus after 2 days of incubation at 28 °C.

## Figures and Tables

**Table 1:** Strains and plasmids used in this study.

Strain or Plasmid	Description	Source or Reference
*S. diastatochromogenes* 1628	Parental strain; toyocamycin producer	Our lab
Δ-*cdgB*	*cdgB* deletion mutant	This study
O-*cdgB*	*cdgB* overexpression mutant	This study
*Fusarium oxysporum* f. sp. *cucumerinum*	Plant pathogen	CICC 2532
*Rhizoctonia solani*	Plant pathogen	CICC 40529
*E. coli* JM109	Host strain for cloning	Our lab
*E. coli* ET12567(PUZ8002)	Donor strain for conjugation	Our lab
pIB139	Derivative of integrative plasmid pSET152, harboring a *PermE* * promoter	Our lab
pKC1139	*E. coli–Streptomyces* shuttle vector	
pKC1139-*cdgB*	*cdgB* deletion vector based on pKC1139	This study
pIB139-*cdgB*	*cdgB* gene under the control of promoter PermE* in pIB139	This study

* a strong promoter.

## Data Availability

Data and materials can be obtained from the research group upon request.

## Supplementary Materials

The following supporting information can be downloaded at: https://www.mdpi.com/article/10.3390/ijms25073878/s1.
