# Supplementary material for: CdgB Regulates Morphological Differentiation and Toyocamycin Production in Streptomyces diastatochromogenes 1628
Source: Int J Mol Sci. 2024 Mar 30;25(7):3878. doi: 10.3390/ijms25073878 (PMC11012013; doi:10.3390/ijms25073878)
Supplement: Supplementary file 1 [file ijms-25-03878-s001.zip › ijms-2914125-supplementary.pdf]

Supplementary Materials

Table S1 Primers used in this study.

Name	Sequence (5' to 3')	Use
**For gene disruption and overexpression**		
cdgBLF	acgacggccagtgccaagctt tgaacgtcgcccgggtactg	
	*Hind*Ⅲ	Deletion of
cdgBLR	cggagccctatgtccgtcttatgtacgtcgagaagcgccg	cdgB gene
cdgBRF	cggcgcttctcgacgtacataagacggacatagggctccg	
cdgBRR	Atgacatgattacgaattcgatatctgatgatgcggatgaaggcg	
	*Eco*RV	
O-cdgB-F	gccggttggtaggatccacatatgatgtcagccggccgcgcgccg，	
	*Nde*I	overexpression
O-cdgB-R	atgacatgattacgaattcgatatcgagaccgagtcggagccctatgtccgtcttgc，	of *cdgB* gene
	*Eco*RV	
**For qRT-PCR**		
RT-toyA-F	GAGAAGCAGATCCTCATCG	*toyA* ORF
RT-toyA-R	ACTGGACAGGTGGAGTTC	
RT-toyM-F	CGGCGACCACTTCATCTA	*toyM* ORF
RT-toyM-R	CCCTTGTAGCAGGACCAG	
RT-toyI-F	GTGATCGAGGACCAGTAG	*toyI* ORF
RT-toyI-R	CCTGTTGGACTTCTACGA	
RT-toyH-F	GATGCGGAAGTCCTCGAT	*toyH* ORF
RT-toyH-R	GGCTGTTGACGATCAGTT	
RT-toyG-F	GAAGACCAGCACCACCTC	*toyG* ORF
RT-toyG-R	CGGCACCTATCCCTACAC	
RT-toyF-F	TCAACTGGTCGAGGAAC	*toyF* ORF
RT-toyF-R	GGTGATGATCGGACGGAC	
RT-toyE-F	CTCGATCAGGTCCCTCGT	*toyE* ORF
RT-toyE-R	ACCGACGACTATCTGGAG	
RT-toyD-F	GAGACCATGACCAGTTGT	*toyD* ORF
RT-toyD-R	AAGCTGCTGATCGAGATC	
RT-toyC-F	CAATCGAGTGGTCAGGTT	*toyC* ORF
RT-toyC-R	CGCCTTCAAGTTCGTCTG	
RT-toyB-F	CAGTTCGACGACGTAGTT	*toyB* ORF
RT-toyB-R	TCACCAAAGAGTTCCACTT	

#The restriction sites are shown underlined.
